# Droplet digital PCR-based *EGFR* mutation detection with an internal quality control index to determine the quality of DNA

**DOI:** 10.1038/s41598-017-18642-x

**Published:** 2018-01-11

**Authors:** Sung-Su Kim, Hyun-Jeung Choi, Jin Ju Kim, M. Sun Kim, In-Seon Lee, Bohyun Byun, Lina Jia, Myung Ryurl Oh, Youngho Moon, Sarah Park, Joon-Seok Choi, Seoung Wan Chae, Byung-Ho Nam, Jin-Soo Kim, Jihun Kim, Byung Soh Min, Jae Seok Lee, Jae-Kyung Won, Soo Youn Cho, Yoon-La Choi, Young Kee Shin

**Affiliations:** 1 Laboratory of Molecular Pathology and Cancer Genomics, College of Pharmacy, Seoul National University, Seoul, Korea; 2 The Center for Companion Diagnostics, LOGONE Bio Convergence Research Foundation, Seoul, Korea; 3 The Center for Anti-Cancer Companion Diagnostics, Bio-MAX/N-Bio, Seoul National University, Seoul, Korea; 4 R&D Center, Gencurix Inc., Seoul, Korea; 5 Department of Pharmacology, School of Life Science and Biopharmaceutics, Shenyang Pharmaceutical University, Shenyang, China; 6 CRO Center, Abion Inc., Seoul, Korea; 7 College of Pharmacy, Daegu Catholic University, Gyeongbuk, Korea; 8 Department of Pathology, Kangbuk Samsung Hospital, Sungkyunkwan University School of Medicine, Seoul, Korea; 9 The Institute of Advanced Clinical & Biomedical Research, HERINGS, Seoul, Korea; 10 Department of Internal Medicine, Seoul National University Boramae Medical Center, Seoul, Korea; 11 Department of Pathology, University of Ulsan College of Medicine, Asan Medical Center, Seoul, Korea; 12 Department of Surgery, Yonsei University School of Medicine, Seoul, Korea; 13 Department of Pathology, Samsung Changwon Hospital, Sungkyunkwan University School of Medicine, Changwon, Korea; 14 Department of Pathology, Seoul National University Hospital, Seoul National University College of Medicine, Seoul, Korea; 15 Department of Pathology and Translational Genomics, Samsung Medical Center, Sungkyunkwan University School of Medicine, Seoul, Korea; 16 Department of Molecular Medicine and Biopharmaceutical Science, Graduate School of Convergence Science and Technology, Seoul National University, Seoul, Korea

## Abstract

In clinical translational research and molecular *in vitro* diagnostics, a major challenge in the detection of genetic mutations is overcoming artefactual results caused by the low-quality of formalin-fixed paraffin-embedded tissue (FFPET)-derived DNA (FFPET-DNA). Here, we propose the use of an ‘internal quality control (iQC) index’ as a criterion for judging the minimum quality of DNA for PCR-based analyses. In a pre-clinical study comparing the results from droplet digital PCR-based EGFR mutation test (ddEGFR test) and qPCR-based EGFR mutation test (cobas EGFR test), iQC index ≥ 0.5 (iQC copies ≥ 500, using 3.3 ng of FFPET-DNA [1,000 genome equivalents]) was established, indicating that more than half of the input DNA was amplifiable. Using this criterion, we conducted a retrospective comparative clinical study of the ddEGFR and cobas EGFR tests for the detection of *EGFR* mutations in non-small cell lung cancer (NSCLC) FFPET-DNA samples. Compared with the cobas EGFR test, the ddEGFR test exhibited superior analytical performance and equivalent or higher clinical performance. Furthermore, iQC index is a reliable indicator of the quality of FFPET-DNA and could be used to prevent incorrect diagnoses arising from low-quality samples.

## Introduction

In light of recent advancements in personalized medicine, nucleic acid-based diagnostics will play a pivotal role in implementation of targeted therapies. In non-small cell lung cancer (NSCLC), *EGFR* exon 19 deletion and L858R mutations are predictive biomarkers for EGFR tyrosine kinase inhibitor (TKI) efficacy; both markers are associated with significantly longer progression-free survival (PFS)^1–5^. Thus, accurate detection of *EGFR* mutations is critical for individualized treatment strategies for NSCLC.

Formalin-fixed, paraffin-embedded tissue (FFPET) is the most widely available material for molecular diagnostics and clinical research^6^. However, the FFPET fixation procedure and long-term storage at room temperature causes several kinds of damage to nucleic acids, creating challenges to molecular analyses using FFPET-derived DNA (FFPET-DNA)^7^. First, DNA fragmentation, a common form of DNA damage, is associated with longer storage periods and the low pH of the formalin used in tissue fixation^8^. The small size of DNAs produced by DNA fragmentation in FFPET affect PCR amplification, and the success rate of PCR is dependent on the size of the amplicon^9^. Second, hydrolytic deamination of cytosine yields uracil lesions, which have been identified as a major source of sequence artifacts in FFPET-DNA^10,11^. Among the sequence artifacts detected in FFPET-DNA, C:G > T:A transitions are the most frequent type of single nucleotide variants (SNVs)^12^. Surprisingly, the *EGFR* T790M mutation, which causes TKI resistance in NSCLC patients, can yield a false-positive result owing to cytosine deamination^13,14^. Third, cross-linked DNA reduces the stability of dsDNA, affecting the amount of FFPET-DNA that can be amplified by PCR^15^. Cross-linking not only causes problems with DNA isolation, but also significantly affects PCR amplification^16^. Fourth, formalin fixation time significantly influences the quality of FFPET-DNA and consequently the results of PCR analysis^17^. Thus, because of the differences in storage duration and fixation procedures among laboratories, FFPET-DNA quality should be checked before being used in clinical studies.

Droplet digital PCR (ddPCR) is an assay that combines state-of-the-art microfluidics technology with TaqMan-based PCR to achieve precise target DNA quantification at high levels of sensitivity and specificity. Due to its technological advantages, which confer highly sensitive mutation detection, this method has been adopted in clinical research^18–22^. Notably, DNA integrity can also be assessed using ddPCR technology^19^. However, the clinical performance of ddPCR-based tests with FFPET-DNA quality measurement has not been subjected to a detailed comparison with gold standards, such as qPCR.

In this study, we report the development of sample criteria for the minimum FFPET-DNA quality suitable for PCR, and the application of these criteria to a ddPCR-based EGFR mutation test. To establish our criteria, we collected and analyzed 316 NSCLC FFPET-DNA samples of various ages and qualities from three sites. In addition, we compared the performance of the GenesWell ddEGFR mutation test (ddEGFR test) with that of the cobas EGFR mutation test (cobas EGFR test) in a retrospective clinical study using 171 NSCLC FFPET samples. Collectively, our results indicate that the use of sample criteria is critical for validating performance in clinical studies.

## Results

### Validation of internal quality control (iQC) in the ddEGFR test

Because the ddPCR-based test can lead to false positive results due to its intrinsic high sensitivity and FFPET characteristics, the cut-offs of the ddEGFR test were determined based on false-positive analyses using normal FFPET. Mutation calls were identified based on true-positive mutation values higher than limit of blank (LoB) and limit of detection (LoD) mutation index (MI) (1), which were established from analytical performance studies (Table 1). MI is a numerical value representing the ratio of mutant to internal quality control (iQC) copies, calculated as follows:

1 $${\rm{Mutation}}\,\text{index}\,( \% )=\frac{Mutant\,copies}{iQC\,copies}\,\times \,100$$

**Table 1:** Limit of blank and limit of detection of the ddEGFR test for mutant calls.

Exon	Mutations detected	Mutation report call	LoB copies/MI (%)*	LoD MI (%)^†^
**18**	G719A, G719C, G719S	G719X	5.6/0.22	0.77
**19**	30 deletions	19del	3.0/0.09	0.83
**20**	S768I	S768I	1.5/0.05	0.83
	T790M	T790M	6.8/0.34	0.78
	C797S^§^	C797S	1.6/0.03	0.75
	5 Insertions	E20Ins	1.6/0.06	0.62
**21**	L858R	L858R	1.6/0.03	0.71
	L861Q	L861Q	1.4/0.05	0.74

*LoB = mean _blank_ + 1.645 (SD _blank_)^36^.

^†^LoD MI (%) = mean_low concentration sample_ + 1.645 (SD_low concentration sample_).

^§^Using plasmids containing the non-predominant mutation for analytical performance.

LoB was calculated using 9.9 ng of normal FFPET-DNA. LoD was measured at 6 points (3–0.1%) serially diluted using NSCLC FFPET-DNA (19del, L858R) and reference standard FFPET-DNA (the other mutations) with wild-type FFPE-DNA. LoD was determined by the lowest amount of DNA that gave an EGFR “Mutation Detected” rate of at least 95% for the target mutation.

In the ddEGFR test, iQC copies can be converted to concentration of input DNA using an FFPE reference standard, suggesting that iQC index (2) is a representative index of amplifiable DNA. Because iQC copies were analyzed using 3.3 ng (1,000 genomic equivalents [GE]) of input DNA per reaction well, iQC index was calculated as follows:

2 $${\rm{iQC}}\,{\rm{index}}=\frac{iQC\,copies}{Input\,DNA\,copies}$$

**Figure 1:**
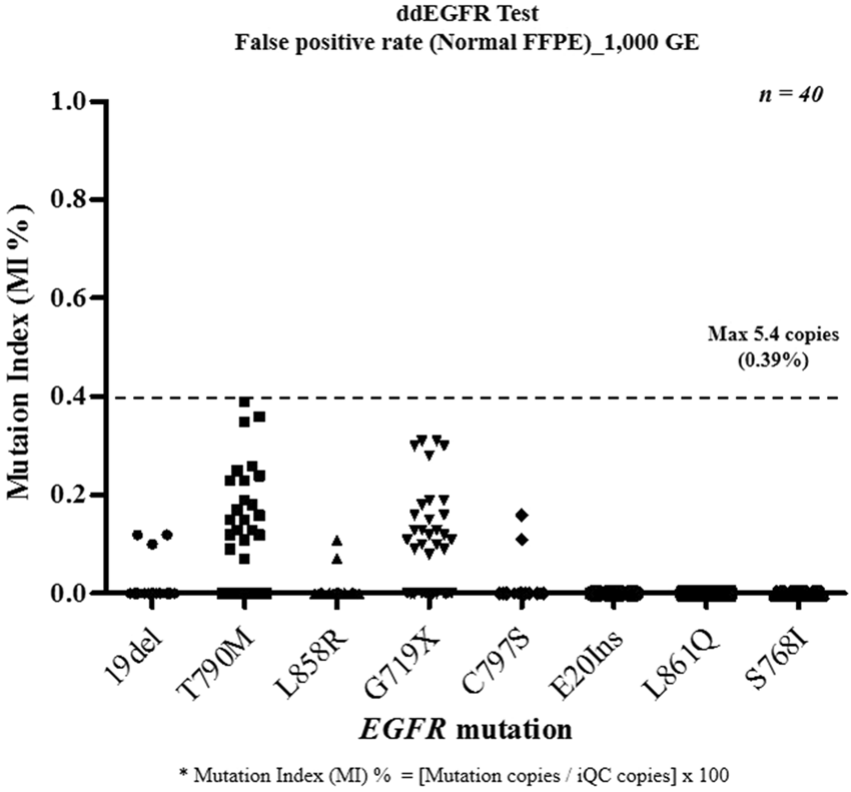
Determination of a suitable cut-off based on false-positive analysis using normal FFPE blocks. Forty normal FFPE specimens were tested, and eight targets were evaluated for each specimen. The maximum number of copies was 5.4 per reaction (black dotted line) and the false-positive rate was below 0.5%.

**Figure 2:**
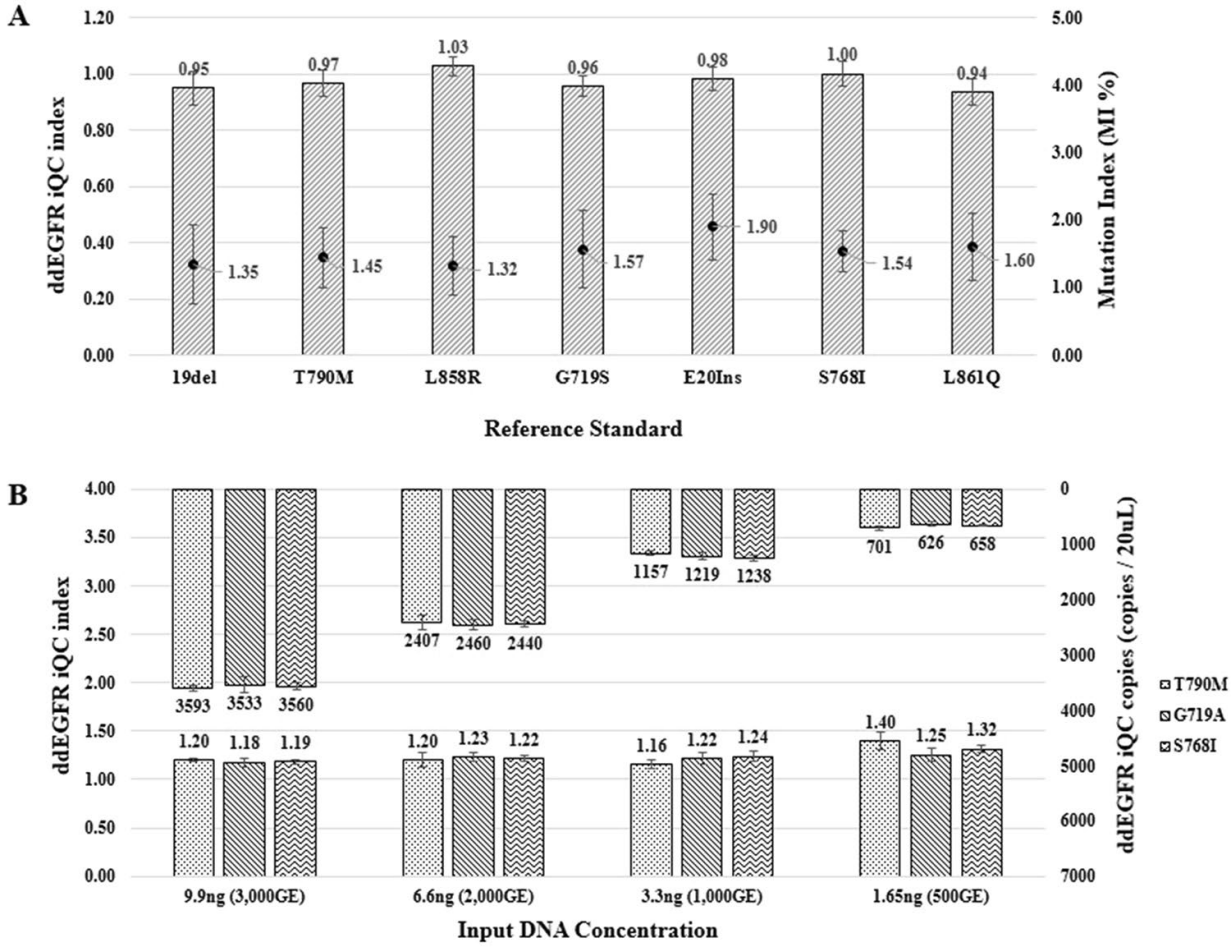
Validation of internal quality control of the ddEGFR test. (**A**) Internal quality control (iQC) validation using reference standard. Each FFPE reference standard DNA extract for the *EGFR* mutations was blended with a fixed amount of wild-type gDNA (3.3 ng, 1,000GE) targeting a 1.5% mutation level, which was validated for use in the ddEGFR test. Values are expressed as the mean ± SD of nine experiments. (**B**) Internal quality control (iQC) validation using the reference standard. Four serial dilutions of each FFPE reference standard DNA extract were prepared and subjected to the ddEGFR test. Error bars indicate SD. Values are expressed as the mean ± SD of three experiments.

Using 40 wild-type FFPET samples, false-positive rates were determined for mutant calling of eight targets. The maximum number of copies was 5.4 per reaction, and the false-positive rates were below 0.5% of MI (Fig. 1). We evaluated iQC using a reference standard. An FFPET-DNA reference standard with *EGFR* mutations was blended with a fixed amount of wild-type gDNA (3.3 ng, 1,000GE) targeting a 1.5% mutation level. The expected iQC index and MI were calculated from the quantity of input DNA, which showed that the measured MI (%) and iQC index closely matched the predicted value (iQC index = 1, MI = 1.5%) (Fig. 2A). Moreover, iQC was also validated by serial dilution of four concentrations of reference standard FFPET-DNA, which revealed that the measured values closely matched the expected values (Fig. 2B) and suggesting that the iQC copies represented the amount of input DNA.

### Comparison of ddEGFR and cobas EGFR tests without sample criteria

**Figure 3:**
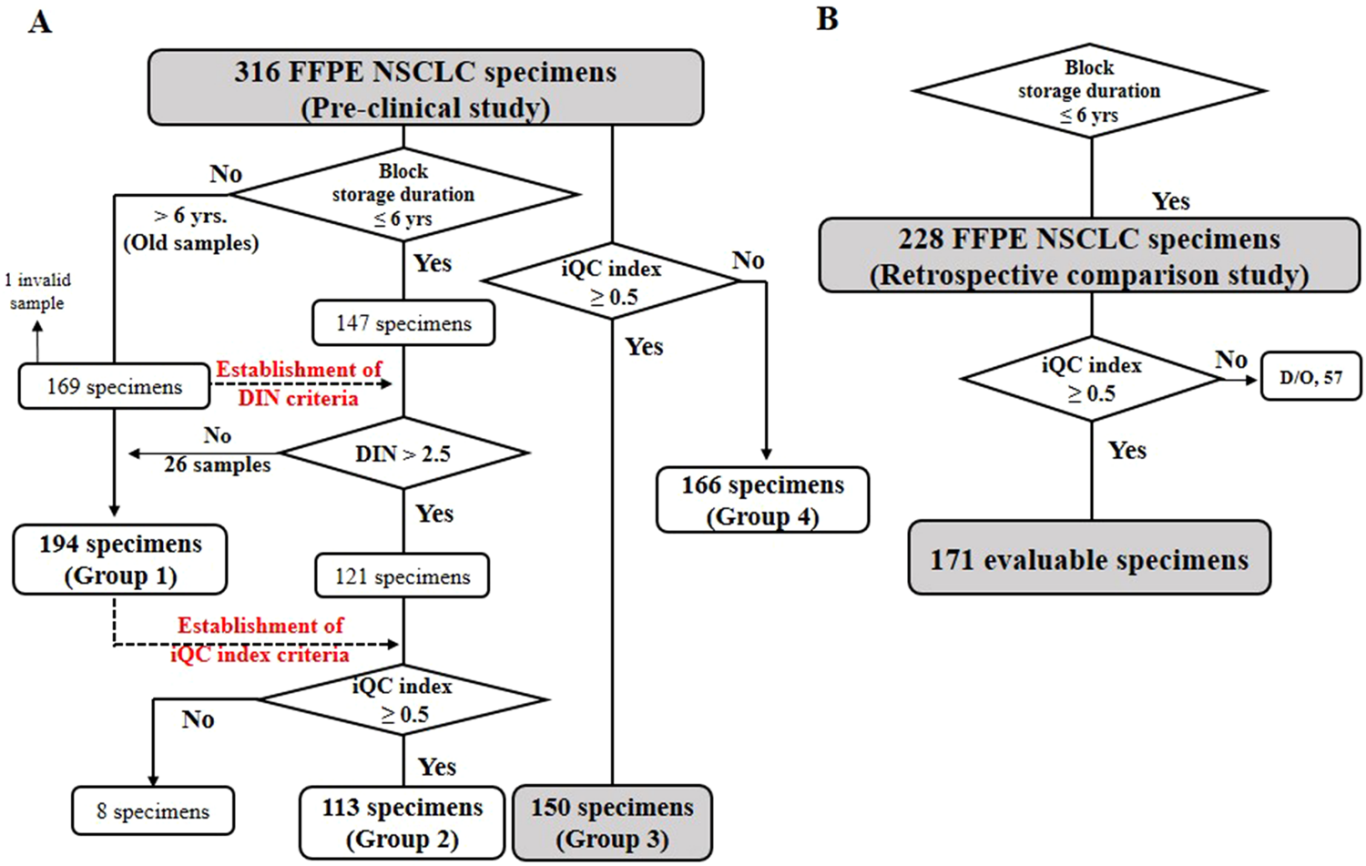
Study design and specimen selection (work flow). (**A**) Patient sample dispositions for the applied sample criteria. For the pre-clinical study group, a total of 316 FFPE specimens were subjected to post-hoc analysis. (**B**) Patient sample dispositions for the applied iQC index. For the retrospective comparison study group, a total of 228 FFPE specimens were analyzed (D/O, Drop Out).

The *EGFR* mutations in 316 NSCLC FFPET samples were analyzed using the ddEGFR and cobas EGFR tests. The study design is depicted in Fig. 3A. Both methods yielded valid results for all but one of the samples. Surprisingly, the ddEGFR and cobas EGFR tests were low concordant (positive percent agreement (PPA) = 94.04%, negative percent agreement (NPA) = 63.41%, overall percent agreement (OPA) = 78.10%, kappa coefficient value (κ) = 0.6650) (see Supplementary Table S2). The cobas EGFR test also exhibited very low concordance with the results of Sanger sequencing (Sanger) of 299 samples (PPA = 59.30%, NPA = 75.00%, OPA = 65.63%, κ = 0.4526) (see Supplementary Table S3).

### Proof-of-concept for determination of minimum DNA quality suitable for PCR using the ddPCR method

**Figure 4:**
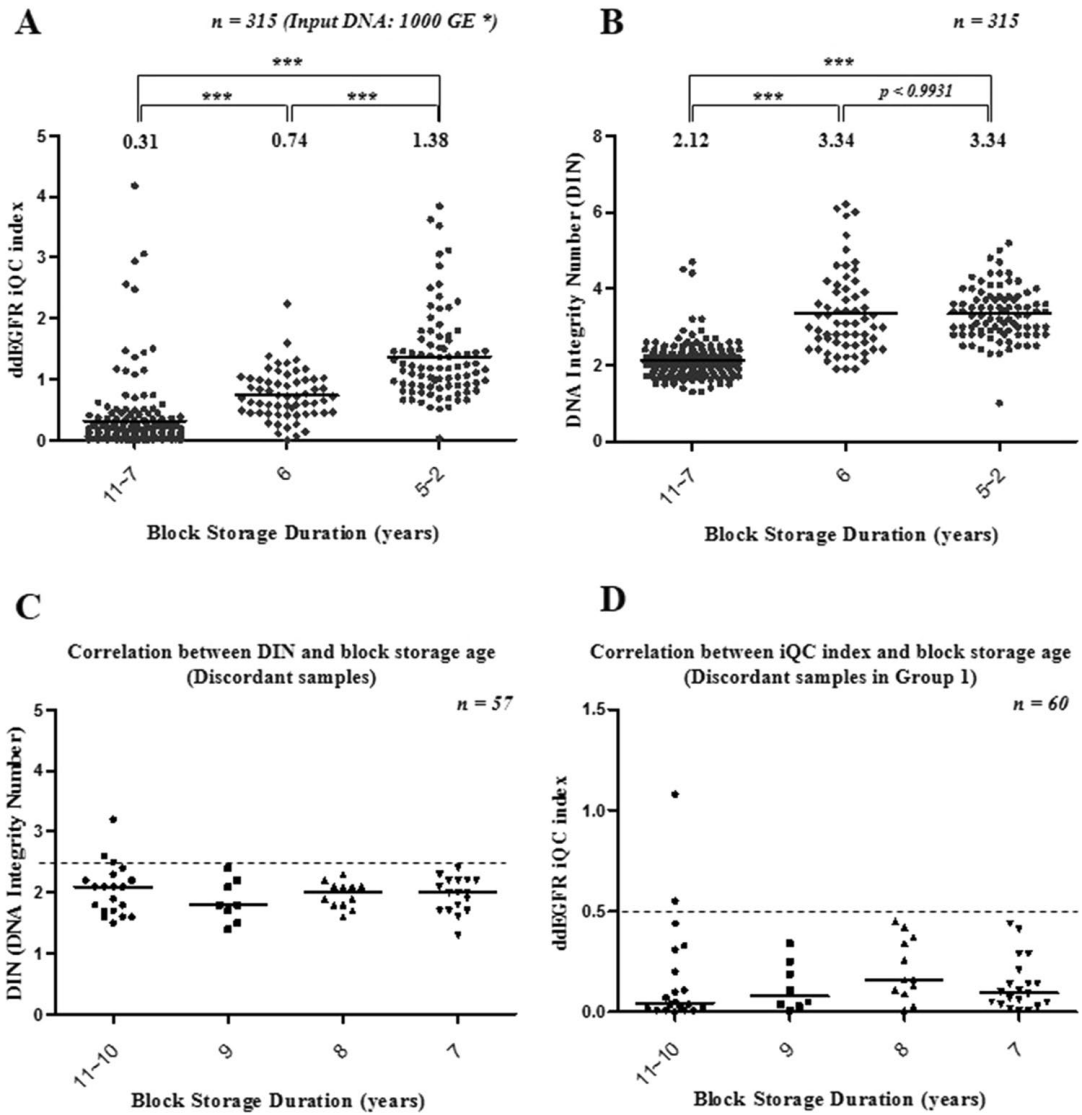
Establishment of sample criteria. (**A**,**B**) Distributions of the ddEGFR iQC index (**A**) and DIN value (**B**) corresponding to sample storage time. The black line indicates the median value (*Genomic Equivalent; ***p < *0.0001*). (**C**,**D**) Establishment of sample criteria using discordant samples. Discordant samples were analyzed by comparing the results of the Cobas EGFR and ddEGFR tests. Plots show distributions of the correlation between DIN value (**C**), iQC index (**D**) and sample storage time of discordant samples. The black line represents the median value. The DIN values and iQC index of discordant samples are distributed under the red dotted line.

To increase the concordance between the ddEGFR and cobas EGFR tests, we investigated the minimum DNA quality suitable for PCR analysis by re-analyzing the ddEGFR data, which provided iQC copies and index (Fig. 2A,B). FFPET block storage duration was reflected in the amount of amplifiable DNA, decreasing below 50% (mean of iQC index = 0.31, standard deviation, SD = 0.57) of 1,000 GE in 7–11-year-old samples. By contrast, in 2–6-year-old samples, the amount of amplifiable DNA was around 100% (mean of iQC index = 1.07, SD = 0.69) (Fig. 4A). Therefore, iQC index decreased with storage duration when FFPET was stored at room temperature. The pattern of DIN values from 315 FFPET-DNA samples measured in parallel was similar to those of iQC copies and index (Fig. 4B).

### Establishment of iQC index for ddEGFR test using FFPET samples

We classified all 316 samples into four groups according to their storage durations, DIN values, and iQC copies, as illustrated in Fig. 1A. First, we analyzed DIN values for 57 discordant samples from among 169 samples derived from blocks stored for more than 6 years. The DIN value was less than 2.5 for most of the discordant samples (Fig. 4C). Among 147 samples with a block storage duration ≤ 6 years, 26 samples did not satisfy our DIN criteria, and were included in Group 1. To establish iQC index criteria, the 60 discordant samples in Group 1 were re-analyzed, and almost all (58/60) had an iQC index < 0.5 (Fig. 4D). Based on this result, the sample criteria were established as follows: block storage duration ≤ 6 years, DIN > 2.5, and iQC index ≥ 0.5. In addition, we observed a strong correlation between ddEGFR iQC index and DIN value, further supporting the idea that iQC index represents the quality of FFPET-DNA (*p* < *0.0001*, see Supplementary Table S4).

### Comparison of ddEGFR and cobas EGFR tests with or without iQC index criteria

When the iQC index cut-off was applied to 121 samples (block storage duration ≤ 6 years, DIN > 2.5), 113 samples remained (Fig. 3A, Group 2). Group 2 samples had a very high concordance rate between the ddEGFR and cobas EGFR tests (PPA = 100.00%, NPA = 76.00%, OPA = 94.69%, κ = 0.9197) (Table 2, see Supplementary Table S5).

**Table 2:** Comparison of ddEGFR and cobas EGFR results from FFPE samples eliminated by the sample criteria.

Pre-clinical study group	cobas EGFR Test							
	Group 2				Group 3			
	*n* = *113*	MD	MND	Total	*n* = *150*	MD	MND	Total
**d**dEGFR Test	MD	88	6	94	MD	106	11*	117
	MND	0	19	19	MND	0	33	33
	Total	88	25	**113**	Total	106	44	**150**
PPA (95% C.I.)	100.0% (95.89–100.0%)				100.0% (96.58–100.0%)			
NPA (95% C.I.)	76.00% (54.87–90.64%)				75.00% (59.66–86.81%)			
OPA (95% C.I.)	94.69% (88.80–98.03%)				92.67% (87.26–96.28%)			
PPV (95% C.I.)	93.62% (86.62–97.62%)				90.60% (83.80–95.21%)			
NPV (95% C.I.)	100.0% (82.35–100.0%)				100.0% (87.26–100.0%)			

*3 samples: cobas EGFR, 19del; ddEGFR, 19del/T790M.

MD, mutation detected; MND, mutation not detected.

Analysis of concordance rates between ddEGFR and cobas EGFR tests when the sample criteria were applied (Group 2 and Group 3). PPA, NPA, and OPA between the ddEGFR and cobas EGFR tests for the detection of *EGFR* mutations were 100%, 76.00%, and 94.69% (Group 2, left panel) and 100%, 75%, and 92.67% (Group 3, right panel), respectively.

To determine the clinical implications of the iQC index, we applied this criterion to 316 FFPETs, resulting in the classification of 150 samples into Group 3 (Fig. 3A), and re-analyzed the concordance rate between the ddEGFR and cobas EGFR results. Group 3 samples had a very high concordance rate (PPA = 100.00%, NPA = 75.00%, OPA = 92.67%, κ = 0.8923) (Table 2, see Supplementary Table S6), similar to the results from Group 2 (applying all criteria). By contrast, Group 4 samples, in which the iQC index criterion was not satisfied, exhibited a very low concordance rate (PPA = 78.57%, NPA = 58.20%, OPA = 63.41%, κ = 0.3862) (see Supplementary Table S7). Therefore, we suggested that iQC index is a key factor in determining whether DNA is of sufficient quality for the ddEGFR test.

### Analysis of discordant samples in a pre-clinical study

Applying the iQC index criterion, we re-analyzed the remaining 11 discordant samples in Group 3. A schematic representation of the re-analysis workflow for discordant samples is depicted in Supplementary Figure S1. In three of the samples, the ddEGFR test reported a double mutation (19del/T790M), whereas the cobas EGFR test and Sanger reported only a single mutation (19del) (Table 2, Group 3). This may be a result of the low detection sensitivity of Sanger (~15%)^23^ and cobas EGFR tests (LoD of T790M = ~3%; cobas EGFR v2). Based on the ddEGFR test results of these three samples, the MI of T790M was ~1% (1.11%, 1.16%, and 1.03%). Additionally, eight discordant samples were verified by Sanger, and no mutations were found (Table 3). Moreover, to observe the effect of tumor ratio, we performed macrodissection to enrich for tumor tissue, and then re-analyzed *EGFR* mutations in eight samples for which the cobas EGFR test had yielded negative results but the ddEGFR test yielded positive results. After macrodissection, the cobas EGFR test gave the same results as the ddEGFR test for four of the eight samples (Table 3). Thus, our results indicate that the ddEGFR test is more sensitive for EGFR mutation detection, independent of tumor ratio. Unusually, one discordant case was a mutation detected (T790M/G719X) in preliminary analysis that was judged invalid by the ddEGFR test after macrodissection. Because the iQC index was very low (0.37, data not shown), it is possible that DNA degradation progress during the macrodissection process.

**Table 3:** Re-analysis of eight of eleven discordant samples (in Group 3).

Sample No	Block Storage age	C/N Ratio (%)	DIN	Preliminary result						After Macrodissection				
				cobas EGFR		ddEGFR		ddEGFR MI (%)	Sanger	cobas EGFR		ddEGFR		ddEGFR MI (%)
1	11	23	3.2	MND	—	MD	L858R	0.8	WT	N/A	N/A	N/A	N/A	—
2	11	51	2.2	MND	—	MD	T790M/G719X	1.08/1.02	Invalid	MND	—	Invalid	—	—
3	6	41	4.6	MND	—	MD	L858R	1.57	WT	MD	L858R	MD	L858R	2.57
4	6	28	3.7	MND	—	MD	G719X	10.17	WT	MD	G719X	MD	G719X	20.17
5	6	12	3.9	MND	—	MD	L858R	5.91	WT	MD	L858R	MD	L858R	4.55
6	6	15	3.1	MND	—	MD	L858R	7.4	WT	N/A	N/A	N/A	N/A	—
7	3	43	4.4	MND	—	MD	20Ins	12.88	WT	MND	—	MD	E20Ins	7.4
8	3	34	4.1	MND	—	MD	19del	15.72	WT	MD	19del	MD	19del	3.15

MD, mutation detected; MND, mutation not detected; N/A, FFPE blocks not available.

Eight of eleven discordant samples were verified by Sanger sequencing. In addition, after increasing the tumor-to-normal tissue ratio, detection of *EGFR* mutations was re-analyzed using the cobas EGFR and ddEGFR tests. After enrichment of tumor tissue, the cobas EGFR test could identify previously undetected *EGFR* mutations in four out of the eight samples. However, the ddEGFR analysis yielded data identical to the preliminary results.

### Retrospective comparative clinical study of EGFR tests

Next, we analyzed the *EGFR* mutation status of 228 samples using the ddEGFR and cobas EGFR tests; 57 samples were excluded based on the iQC index. The study design is depicted in Fig. 3B. The remaining 171 samples with iQC index ≥ 0.5 gave PPA of 98.23%, NPA of 82.76%, and OPA of 92.98% between the ddEGFR and cobas EGFR tests (κ = 0.9029, Table 4, see Supplementary Table S8). Among 12 discordant samples, six were reported to have a double mutation according to the ddEGFR test but only a single mutation according to the cobas EGFR test. As expected, the MI of the additional detected mutation was very low. One discordant case was a mutation not detected by the cobas EGFR test, but detected (L861Q) by both ddEGFR test and Sanger. Conversely, another discordant case was a mutation not detected by the ddEGFR test but detected (19del) by the cobas EGFR test and Sanger (see Supplementary Table S9). This was a rare mutation of the 19del subtype (c.2239_2264del_insGCGAA) caused by a non-specific reaction that is not designed in the cobas EGFR test, and thus it cannot be employed to discriminate the possibility potential erroneous detection^24^ and beneficial cross-reaction of commercial diagnostic kits.

**Table 4:** Retrospective comparative study.

Retrospective comparison study, Applied iQC index (*n* = *171*)		cobas EGFR Test		
		MD	MND	Total
ddEGFR Test	MD	111	10*	121
	MND	2	48	50
	Total	113	58	**171**
PPA (95% C.I.)	98.23% (93.75–99.78%)			
NPA (95% C.I.)	82.76% (70.57–91.41%)			
OPA (95% C.I.)	92.98% (88.06–96.32%)			
PPV (95% C.I.)	91.74% (85.33–95.97%)			
NPV (95% C.I.)	96.00% (86.29–99.51%)			

MD, mutation detected; MND, mutation not detected.

*3 samples: cobas EGFR, 19del; ddEGFR, 19del/T790M.

1 sample: cobas EGFR, G719X; ddEGFR, G719X/L861Q.

1 sample: cobas EGFR, L858R; ddEGFR, G719X/L858R.

1 sample: cobas EGFR, L858R; ddEGFR, T790M/L858R.

Method correlation between ddEGFR and cobas EGFR test. Samples with valid ddEGFR and cobas EGFR test results were included in the agreement analysis. PPA, NPA, and OPA between the ddEGFR and cobas EGFR tests for the detection of *EGFR* mutations were 98.23%, 82.76%, and 92.98%, respectively.

In addition, we measured DIN values from 228 FFPET-DNA samples and observed a pattern similar to that of iQC index. Furthermore, the majority of the most recent samples (within 1 year) had DIN > 2.5 and iQC index ≥ 0.5 (see Supplementary Fig. S2). These data revealed that iQC index is a very powerful indicator of the quality of FFPET-DNA. In addition, these observations demonstrate that the ddEGFR test is a robust diagnostic tool for the accurate detection of *EGFR* mutations in clinical practice.

## Discussion

The quality of FFPET-DNA has been largely ignored in the clinical research and diagnosis field, and the internal controls of most commercial diagnostic kits have been used only to validate assays. Changes in mutation status due to the low quality of FFPET-DNA may result in incorrect diagnoses. Therefore, considerable effort is required to optimize the sample criteria for determining the quality of FFPET-DNA that is suitable for PCR. In this study, we established iQC index criteria to determine the minimum quality of FFPET-DNA and demonstrated the benefits of implementing these criteria benefits in a real-world clinical application.

In our experiments, we found that both automated tissue preparation system (TPS; Siemens Healthcare, Erlangen, Germany), which can minimize handling errors and decrease the effect of formaldehyde-induced DNA–DNA and DNA–protein crosslinks^6,9^, and uracil-DNA glycosylase (UDG) treatment are powerful strategies for reducing false positives caused by sequence artifacts^10,25–27^. However, sequence artifacts due to DNA fragmentation remained a problem. Depending on the degree of fragmentation, the same quantities of DNA from different FFPET samples can contain widely different amounts of amplifiable DNA templates^28^. For this reason, PCR-based methods such as qPCR, ddPCR, and next-generation sequencing (NGS) are preferable for quantifying the amount of amplifiable template in FFPET-DNA^19^.

We showed that iQC copies can be used to as an indicator of the concentration of input DNA (Fig. 2A,B) and that a novel concept for mutation calling termed MI can be used as an indicator of the mutation level, which in turn reflects DNA quality. MI provides a much more accurate indication of the mutation level than mutation frequency, which is simply calculated based on input DNA concentration. Accordingly, we designed an iQC that measures the amount of *EGFR* exon 20 to assess the amount of amplifiable FFPET-DNA. Because elevated *EGFR* expression has been observed in NSCLC^23^, the internal control should be designed to represent the *EGFR* gene copy number.

Interestingly, some discordant samples reported as single mutations by the cobas EGFR test were shown to have double mutation by the ddEGFR test in pre-clinical (Table 2) and clinical studies (Table 4). The additional detected MI was around 1%, as expected. According to a clonal selection model, EGFR-TKI treatment may lead to the selection of T790M mutant cells, and thus even a small fraction of T790M positive tumor cells at the beginning of treatment could lead to clinical EGFR-TKI resistance^16^. Therefore, is very important to detect even a small fraction of mutations.

The discordant results were largely due to the difference in LoD between both tests. Specifically, some mutations detected by the ddEGFR test cannot be detected by the cobas EGFR test because the sensitivity of the ddEGFR test is higher. In the pre-clinical study Group 3, L858R MIs were relatively low in cobas EGFR-negative samples (around 5%, Table 3). Considering that the LoD of the cobas EGFR test was 5%, these MI values, which reflect sample quality and mutation level, are reasonable. When the iQC index was applied, the proportion of such discordant samples was small.

The cobas EGFR test recommends macrodissection of low-percent tumor tissues (below 10%) to improve detectability^29^. When the cobas EGFR test yielded a result of ‘mutation not detected’ (MND), even though the C/N ratio was over 10%, the *EGFR* mutation was re-analyzed after increasing the tumor ratio by macrodissection. The cobas EGFR test yielded the same results as the ddEGFR test for four of the eight samples (Table 3), showing that ddEGFR exhibits superior analytical performance. Thus, the detectability of the cobas EGFR test may be increased by enriching for tumor tissue, e.g., by macrodissection, but this manipulation requires extra time and effort. By contrast, the ddEGFR test does not require macrodissection and improves reliability.

For 1-year-old samples (*n* = *46*) in the clinical study group, all iQC index and DIN values satisfied the sample criteria (iQC index ≥ 0.5, DIN > 2.5) (see Supplementary Fig. S2), suggesting that the low age samples used in clinical practice will have minimal problems with DNA quality. Previous studies have reported that cobas EGFR test and Sanger sequencing results are highly concordant when used on low age samples^30–32^. By contrast, the concordance rate between cobas EGFR test and Sanger sequencing of pre-clinical study samples was very low (see Supplementary Table S3), but was improved when the iQC index was applied (Data not shown). Given the additional cost and effort required for DIN measurement, the iQC index is a robust indicator of the minimum DNA quality required for PCR amplification, as it takes into account the various types of DNA damage caused by FFPET processing and long-term storage.

In addition, iQC index ≥ 0.5 is an indicator that guarantees clinical equivalence obtained through comparative clinical studies with existing approved diagnostics test (cobas EGFR test). Clinical equivalence cannot be guaranteed for samples with an iQC index < 0.5. However, discordance of samples with iQC index < 0.5 can be explained by two causes. The first cause may be due to the possibility of false negative results of cobas EGFR test. Second, there is a possibility of false positive result due to ultra-sensitivity of ddEGFR test. The analytical performance of the ddEGFR test indicates a high possibility of false negative results on the cobas EGFR test. However, it is questionable whether mutations detected in samples with an iQC index < 0.5 (especially mutations detected at low MI %) are related to drug treatment response. This must be verified through prospective clinical trials. Small-scale independent retrospective clinical studies for the ddEGFR test have shown responses of patients treated with an EGFR-TKI, even of patients with an iQC index < 0.5 (unpublished). Therefore, an iQC index ≥ 0.5 is a numerical value used in comparative clinical studies to guarantee clinical equivalence between the results of the ddEGFR and cobas EGFR tests. This means that iQC index may be changed through an independent prospective clinical trial for ddEGFR test. Also, using another approved kit for comparative clinical study, the iQC index may be changed for clinical equivalence. This is the result of considering clinical cut-off not only to compare the mutation results but also to ensure EGFR-TKI response.

We also observed that the iQC index could be used to obtain a diagnosis using other cancer type samples (colorectal cancer) and other diagnostic kits, including the ddPCR-based KRAS mutation test and the cobas KRAS mutation test. Similar to the results of this study, we observed that when the iQC index was applied, the concordance rate between the results of the two kits increased significantly (data not shown). Overall, the iQC index could be a useful criterion for judging the quality of FFPET-DNA.

In particular, the iQC index seems to be more useful in diagnoses using liquid biopsy samples (e.g. cell free tumor DNA, ctDNA). The iQC index may be necessary for liquid biopsy specimens since the quality of ctDNA can vary diversely by frequent fragmentation, which is a biological property of ctDNA, variations in sample collection and storage by each hospitals, and by logistic issues.

In conclusion, detection of genetic mutations in FFPET samples is difficult due to DNA fragmentation during the storage period and sequence artifacts arising from DNA damage. The iQC index allowed selection of appropriate FFPET-DNA samples for companion diagnosis using a ddPCR-based mutation test. Furthermore, we suggest that clinical trials using FFPE should present criteria reflecting the quality of the DNA. Our ddPCR-based EGFR mutation test exhibited superior analytical performance to the cobas EGFR test. A future clinical study should evaluate the use of the ddPCR-based EGFR test to determine the suitability of NSCLC patients for EGFR-TKI treatment in cases in which the cobas EGFR test reports no mutation.

## Materials and Methods

### Study design

To establish sample criteria, a total of 316 samples obtained from NSCLC patients were tested for *EGFR* mutations. A post-hoc analysis of these pre-clinical data was conducted for both the ddEGFR and cobas EGFR test results. Based on the established sample criteria, an independent retrospective comparison study was performed to estimate the concordance between the ddEGFR and cobas EGFR tests; for this purpose, 228 FFPET-DNA samples from NSCLC patients were analyzed by both tests. Both EGFR mutation tests were performed in a double-blind fashion by an independent laboratory (Abion Inc., Seoul, Korea). The study design (workflow) is depicted in Fig. 3.

The major study objectives were 1) to establish sample criteria to determine the minimum FFPET-DNA quality suitable for PCR, and 2) to compare the clinical performances of the ddEGFR and cobas EGFR tests.

### FFPET collection and DNA extraction, and determination of DNA quantity and quality

FFPET blocks of resected or biopsy samples from NSCLC patients (*n* = 316) collected from 2005 to 2014 were retrieved from the Department of Pathology, Samsung Medical Center (*n* = *200*) (SMC; Seoul, Korea), Asan Medical Center (*n* = *66*) (AMC; Seoul, Korea), and Severance Hospital (*n* = *50*) (Seoul, Korea). This study was approved by the Institutional Review Board (IRB) of SMC and Seoul National University (study ID: SMC-2014-05-084-002). For the retrospective comparison study, 228 archived FFPET blocks from NSCLC patients collected between 2010 and 2016 were obtained from Department of Pathology, SMC. This study was approved by the IRB of SMC and the Ministry of Food and Drug Safety (MFDS) of Korea (study ID: SMC-2016-07-104-002). Patient information was anonymized and de-identified prior to analysis. From each FFPET, 10 μm sections were cut and subjected to DNA extraction. H&E-stained sections containing tumor lesions marked by a pathologist (S.W.C.) were scanned, and the cancer/normal (C/N) ratio was calculated using the Pannoramic Viewer Software v.1.15.4 (3DHISTECH, Budapest, Hungary). DNA extraction from FFPETs was performed using an automated Tissue Preparation System (TPS; Siemens Healthcare, Erlangen, Germany) with VERSANT^®^ Tissue Preparation Reagents, as described previously^33^. Total nucleic acids were eluted with 100 μL elution buffer containing UDG provided by the manufacturer. For all samples, DNA concentration was assessed using the Qubit™ 3.0 Fluorometer (ThermoFisher Scientific, MA, USA). The DNA integrity number (DIN), reflecting the DNA fragmentation level^34^ of genomic DNA (gDNA), was analyzed on a 2200 TapeStation system with Genomic DNA Screen Tape (Agilent Technologies, CA, USA).

### Validation of internal quality control (iQC) of ddEGFR test

The ddEGFR test (Gencurix Inc., Seoul, Korea) was designed as a highly sensitive ddPCR-based diagnostic test for detecting 45 mutation sites within the exon 18–21 region of the *EGFR* gene using four reactions. The amplified fragments, which contain the fluorophores FAM™ or HEX™, are displayed as dots (droplets) and can be used to calculate concentrations (copies/20 μL) based on the Poisson distribution^35^. The details of the specific mutations detected by the assay are provided in Table 1 and Supplementary Table S1. The non-clinical performance studies followed the guidelines approved by the Clinical and Laboratory Standards Institute (CLSI) and the Korea-MFDS. For validation of internal quality control of ddEGFR test, FFPE reference standard DNA extracts (HDx™ Reference Standard, Horizon Discovery, Cambridge, UK) whit EGFR mutations were blended with a fixed amounts of wild-type gDNA (3.3 ng, 1,000 GE; Promega, Fitchburg, WI, USA) and each sample, with a target MI of 1.5%. In addition, four serial dilutions of each sample (9.9 ng, 6.6 ng, 3.3 ng, and 1.65 ng) were prepared and analyzed using the ddEGFR test. The iQC copies and target MI of each sample was confirmed based on the input DNA concentration and target MI (1.5%).

### Biomarker analysis

The ddEGFR test was performed in a 20 μL volume containing 3.3 ng (1,000 GE)/reaction of template DNA on a Droplet Digital™ PCR (ddPCR) system (Bio-Rad, Hercules, CA, USA). The ddPCR assay was conducted as described previously^33^. Thresholds for detection were set manually based on results from non-template control wells and negative control wells containing wild-type gDNA (Promega). PCR amplification for the cobas EGFR test (Roche Molecular Systems Inc., Branchburg, NJ, USA) was performed on a cobas z 480 Analyzer. The cobas EGFR test requires 150 ng total input DNA. Both mutation tests were analyzed in a double-blind fashion, and the results were matched after analysis.

For mutation screening of *EGFR* exons 18, 19, 20, and 21 by 2× bidirectional Sanger sequencing, regions of interest were amplified by PCR, and the amplified samples were processed at an independent laboratory (Macrogen, Seoul, Korea) using a validated protocol. Sanger sequencing results were cross-checked and interpreted by a pathologist (Y.L.C.).

### Methods correlation and statistical analysis

Agreement analysis for all methods was based on mutation report calls (Table 1). Statistical analysis was performed using GraphPad Prism™ (GraphPad Software Inc., San Diego, USA) and the R 1.6.12 package ‘psych’ (http://CRAN.R-project.org/package=psych). For the agreement analyses, positive percent agreement (PPA), negative percent agreement (NPA), and overall percent agreement (OPA) were calculated with their corresponding 95% confidence intervals (CIs).

## Electronic supplementary material

Supplemental information
