# Supplementary material for: Droplet digital PCR-based EGFR mutation detection with an internal quality control index to determine the quality of DNA
Source: Sci Rep. 2018 Jan 11;8:543. doi: 10.1038/s41598-017-18642-x (PMC5765147; doi:10.1038/s41598-017-18642-x)
Supplement: Supplementary file 1 — Supplemental information [file 41598_2017_18642_MOESM1_ESM.pdf]

Droplet digital PCR-based *EGFR* mutation detection with an internal quality control index to determine the quality of DNA

Sung-Su Kim, Hyun-Jeung Choi, Jin Ju Kim, M. Sun Kim, In-Seon Lee, Bohyun Byun, Lina Jia, Myung Ryurl Oh, Youngho Moon, Sarah Park, Joon-Seok Choi, Seoung Wan Chae, Byung-Ho Nam, Jin-Soo Kim, Jihun Kim, Byung Soh Min, Jae Seok Lee, Jae-Kyung Won, Soo Youn Cho, Yoon-La Choi, Young Kee Shin

Corresponding author: Young Kee Shin (ykeeshin@snu.ac.kr)

Supplementary Tables

Supplementary Table. S1. *EGFR* mutation detected by the ddEGFR mutation test.

No.	Target exon	COSMIC No. *	Target mutant A.a	Target mutant site	Result
1		6239	p.G719A	c.2156G>C	
2	**Exon18**	6252	p.G719S	c.2155G>A	**G719X**
3		6253	p.G719C	c.2155G>T	
4		6223	p.E746_A750del	c.2235_2249 del 15	
5		13551	p.E746_T751>I	c.2235_2252 > AAT	
6		12728	p.E746_T751del	c.2236_2253 del 18	
7		12678	p.E746_T751>A	c.2237_2251 del 15	
8		12367	p.E746_S752>A	c.2237_2254 del 18	
9		12384	p.E746_S752>V	c.2237_2255>T	
10		6225	p.E746_A750del	c.2236_2250 del 15	
11		6220	p.E746_S752>D	c.2238_2255 del 18	
12		13550	p.E746_A750>IP	c.2235_2248>AATTC	
13		12403	p.L747_S752>Q	c.2239_2256>CAA	
14		12422	p.L747_A750>P	c.2238_2248 >GC	
15		12419	p.L747_T751>Q	c.2238_2252 >GCA	
16		6218	p.L747_E749del	c.2239_2247 del 9	
17	**Exon19**	12382	p.L747_ A750>P	c.2239_2248 TTAAGAGAAG>C	**19del**
18		6210	p.L747_T751>S	c.2240_2251 del 12	
19		12383	p.L747_T751>P	c.2239_2251>C	
20		13552	p.E746_T751>IP	c.2235_2251>AATTC	
21		6254	p.L747_T751del	c.2239_2253 del 15	
22		6255	p.L747_S752del	c.2239_2256 del 18	
23		12387	p.L747_P753>Q	c.2239_2258 >CA	
24		12370	p.L747_P753>S	c.2240_2257 del 18	
25		12416	p.E746_T751>VA	c.2237_2253>TTGCT	
26		-	-	c.2239_2257>GT	
27		26038	p.K745_E749del	c.2233_2247del15	
28		13556	p.S752_I759del	c.2253_2276del24	
29		12386	p.E746_T751>V	c.2237_2252>T	
30		12385	p.E746_S752>I	c.2235_2255>AAT	
31		18427	p.E746_P753>VS	c.2237_2257>TCT	

32		12369	p.L747_T751del	c.2240_2254 del 15	
33		23571	p.L747_T751delLREAT	c.2238_2252 del 15	
34	**Exon20**	12376	p.V769_D770insASV	c.2307_2308 insGCCAGCGTG	**E20Ins**
35		12377	p.H773_V774insH	c.2319_2320 insCAC	
36		12378	p.D770_N771insG	c.2310_2311 insGGT	
37		13428	p.D770_N771insSVD	c.2311_2312insGCGTGGACA	
38		13558	p.V769_D770insASV	c.2309_2310AC>CCAGCGTGGAT	
39	**Exon20**	6241	p.S768I	c.2303G>T	**S768I**
40		6240	p.T790M	c.2369C>T	**T790M**
41		-	p.C797S	c.2389T>A	**C797S**
42		-	p.C797S	c.2390G>C	
43	**Exon21**	6224	p.L858R	c.2573T>G	**L858R**
44		12429	p.L858R	c.2573_2574TG>GT	
45		6213	p.L861Q	c.2582T>A	**L861Q**

* Catalogue of Somatic Mutations in Cancer (COSMIC). 2016, v.78.
http://cancer.sanger.ac.uk/cosmic

Forty-five *EGFR* mutations were detected by the ddEGFR mutation test.

Supplementary Table. S2. Concordance rates for the detection of the *EGFR* mutations between the ddEGFR test and cobas EGFR test using evaluable specimens (*n = 316*, without application of sample criteria).

**All Samples (*n = 316*)**		**cobas EGFR Test**		
		**MD**	**MND**	**Total**
**ddEGFR Test**	**MD**	139	62 *	201
	**MND**	9	104	113
	**Total**	148	166	**314**
**PPA (95% C.I.)**	94.04% (88.99–97.24%)			
**NPA (95% C.I.)**	63.41% (55.55–70.79%)			
**OPA (95% C.I.)**	78.10% (73.12–82.54%)			
**PPV (95% C.I.)**	70.30% (63.48–76.51%)			
**NPV (95% C.I.)**	92.04% (85.42–96.29%)			

1 Sample: Invalid

* 3 samples: cobas EGFR, 19del; ddEGFR, 19del/T790M

1 sample: cobas EGFR, 19del; ddEGFR, 19del/L858R

1 sample: cobas EGFR, E20Ins; ddEGFR, E20Ins/T790M

1 sample: cobas EGFR, S768I; ddEGFR, S768I/L858R

All Samples (*n = 316*)		cobas EGFR Test														
		**G719X**	**19del**	**T790M**	**E20Ins**	**S768I**	**L858R**	**G719X, T790M**	**G719X, S768I**	**19del, T790M**	**19del, L858R**	**T790M, L858R**	**T790M, E20Ins**	**S768I, L858R**	**MND**	**Total**
**ddEGFR Test**	**G719X**	1													1	2
	**19del**		85												4	89
	**T790M**			0												0
	**E20Ins**				0										2	2
	**S768I**					0										0
	**L858R**		1 †				48								47	96
	**G719X,T790M**							0							1	1
	**G719X,S768I**								3							3
	**19del,T790M**		3							0						3
	**19del,L858R**		1								0					1
	**T790M,L858R**											0			1	1
	**T790M,E20Ins**				1								0			1
	**S768I, L858R**					1								2		3
	**MND**		9												104	113
	**Total**	1	99	0	1	1	48	0	3	0	0	0	0	2	160	315

1 Sample: Invalid

† 1 sample was excluded from the analysis

κ coefficient = 0.6650 (95% C.I. 59.98–73.03%)

A total of 316 samples with valid ddEGFR and cobas EGFR test results were included in the agreement analysis. PPA, NPA, and OPA between the ddEGFR and cobas EGFR tests for the detection of *EGFR* mutations were 94.04%, 63.41%, and 78.10%, respectively.

MD, mutation detected; MND, mutation not detected.

Supplementary Table. S3. Sanger sequencing versus the cobas EGFR test for the detection of *EGFR* mutations in evaluable specimens (*n* = *299*)

W/O Criteria(*n* = *299*)		Sanger sequencing		
		**MD**	**MND**	**Total**
**cobas EGFR test**	**MD**	102	29 *	131
	**MND**	70 †	87	157
	**Total**	172	116	**288**
**PPA (95% CI)**	59.30% (61.56–66.72%)			
**NPA (95% CI)**	75.00% (66.11–82.57%)			
**OPA (95% CI)**	65.63% (59.83–71.10%)			
**PPV (95% CI)**	77.86% (69.78–84.65%)			
**NPV (95% CI)**	55.41% (47.28–63.34%)			

* 1 sample: Sanger, L858R; cobas EGFR, L858R, S768I

† 4 samples: Sanger, 19del, L858R; cobas EGFR, 19del

1 sample: Sanger, 19del, L858R; cobas EGFR, L858R

W/O Criteria (*n = 299*)		Sanger Sequencing														
		**G719X**	**19del**	**T790M**	**E20Ins**	**S768I**	**L858R**	**19del,G719X**	**19del, T790M**	**19del, L858R**	**G719X, T790M**	**G719X, S768I**	**T790M, L858R**	**S768I, L858R**	**MND**	**Total**
**cobas EGFR test**	**G719X**	0													1	1
	**19del**		62				8 §			4					18	92
	**T790M**			0												0
	**E20Ins**				0										1	1
	**S768I**					0										0
	**L858R**		2 §				40			1					5	48
	**19del,G719X**							0								0
	**19del, T790M**								0							0
	**19del, L858R**									0						0
	**G719X,T790M**										0					0
	**G719X,S768I**		1 §									0			2	3
	**T790M,L858R**												0			0
	**S768I, L858R**						1							0	1	2
	**MND**		24				27	1		13					87	152
	**Total**	0	89	0	0	0	76	1	0	18	0	0	0	0	115	299

§ 11 samples were exclude from the analysis

κ coefficient = 0.4526 (95% C.I. 37.45–53.07%)

Concordance of *EGFR* mutation detection between the cobas EGFR test and Sanger sequencing, without application of exclusion criteria. A total of 299 samples with valid Sanger sequencing and cobas EGFR test results were included in the analysis. Seventeen samples could not be evaluated for concordance because Sanger sequencing did not yield results. MD, mutation detected; MND, mutation not detected.

Supplementary Table. S4. Correlation between ddEGFR iQC index and DIN value.

	iQC index \ DIN	Bad (< 2.5)	Good (> 2.5)
**Group 1 (*n = 194*)** (*p. < 0.0001*)	**Low (< 0.5)**	155	2
	**High (≥ 0.5)**	22	15
**Group 2 (*n = 113*)**	**Low (< 0.5)**	0	8
	**High (≥ 0.5)**	0	113
**Group 3 (*n = 150*)**	**Low (< 0.5)**	0	0
	**High (≥ 0.5)**	22	128
**Group 4 (*n = 166*)**	**Low (< 0.5)**	156	10
	**High (≥ 0.5)**	0	0

The ddEGFR iQC index and DIN value of excluded samples were lower than the values of the sample criteria. Results from included samples were the opposite to those of the excluded samples. In addition, the ddEGFR iQC index and DIN values were strongly correlated.

Supplementary Table. S5. Comparison of ddEGFR and cobas EGFR results, with application of sample criteria (detailed concordance rates between ddEGFR and Cobas EGFR test results).

**Group 2 (*n* = *113*)**		**cobas EGFR Test**										
		**G719X**	**19del**	**T790M**	**E20Ins**	**S768I**	**L858R**	**G719X, T790M**	**G719X, S768I**	**S768I, L858R**	**MND**	**Total**
**ddEGFR Test**	**G719X**	1									1	2
	**19del**		51								1	52
	**T790M**			0								0
	**E20Ins**				0						1	1
	**S768I**					0						0
	**L858R**						33				3	36
	**G719X, T790M**							0				0
	**G719X, S768I**								2			2
	**S768I, L858R**									1		1
	**MND**										19	19
	**Total**	1	51	0	0	0	33	0	2	1	25	**113**

κ coefficient = **0.9197** (95% C.I. 85.85–98.09%)

Comparison between the ddEGFR and cobas EGFR test results with application of the sample criteria (Group 2). MND, mutation not detected.

Supplementary Table. S6. Comparison of ddEGFR and cobas EGFR results, with application of the iQC index (detailed concordance rates between ddEGFR and cobas EGFR test results).

Group 3 (*n = 150*)		cobas EGFR Test											
		**G719X**	**19del**	**T790M**	**E20Ins**	**S768I**	**L858R**	**G719X, T790M**	**G719X, S768I**	**19del,T790M**	**S768I, L858R**	**MND**	**Total**
**ddEGFR Test**	**G719X**	1										1	2
	**19del**		61									1	62
	**T790M**			0									0
	**E20Ins**				0							1	1
	**S768I**					0							0
	**L858R**						41					4	45
	**G719X, T790M**							0				1	1
	**G719X, S768I**								2				2
	**19del,T790M**		3							0			3
	**S768I, L858R**										1		1
	**MND**											33	33
	**Total**	1	64	0	0	0	41	0	2	0	1	41	150

κ coefficient = **0.8923** (95% C.I. 83.25–95.22%)

Comparison between the ddEGFR and cobas EGFR test results, with application of only the iQC criterion (Group 3). MND, mutation not detected.

Supplementary Table. S7. Analysis of concordance rates using excluded samples (Group 4).

**Group 4 (*n = 166*)**		**cobas EGFR Test**		
		**MD**	**MND**	**Total**
**ddEGFR Test**	**MD**	33	51 *	84
	**MND**	9	71	80
	**Total**	42	122	**164**
**PPA (95% C.I.)**	78.57% (63.19–89.70%)			
**NPA (95% C.I.)**	58.20% (48.93–67.06%)			
**OPA (95% C.I.)**	63.41% (55.55–70.79%)			
**PPV (95% C.I.)**	39.29% (28.80–50.55%)			
**NPV (95% C.I.)**	88.75% (79.72–94.72%)			

1 sample: Invalid

* 1 sample: cobas EGFR, 19del; ddEGFR, 19del/L858R

1 sample: cobas EGFR, E20Ins; ddEGFR, E20Ins/T790M

1 sample: cobas EGFR, S768I; ddEGFR, S768I/L858R

Group 4 (*n = 166*)		cobas EGFR Test														
		**G719X**	**19del**	**T790M**	**E20Ins**	**S768I**	**L858R**	**G719X, T790M**	**G719X, S768I**	**19del, T790M**	**19del, L858R**	**T790M, E20Ins**	**T790M, L858R**	**S768I, L858R**	**MND**	**Total**
**ddEGFR Test**	**G719X**	0														0
	**19del**		24												3	27
	**T790M**			0												0
	**E20Ins**				0										1	1
	**S768I**					0										0
	**L858R**		1 †				7								43	51
	**G719X, 790M**							0								0
	**G719X, S768I**								1							1
	**19del,T790M**									0						0
	**19del,L858R**		1								0					1
	**T790M,E20Ins**				1							0				1
	**T790M,L858R**												0		1	1
	**S768I, L858R**					1								1		2
	**MND**		9												71	80
	**Total**	0	35	0	1	1	7	0	1	0	0	0	0	1	119	165

1 sample: Invalid

† 1 sample was excluded from the analysis

κ coefficient = **0.3862** (95% C.I. 27.85–49.40%)

Re-analysis of concordance rate using excluded samples. Samples were selected by iQC index (< 0.5). The 166 excluded samples with valid ddEGFR and cobas EGFR test results were included in the agreement analysis. PPA, NPA, and OPA between the ddEGFR and cobas EGFR tests for the detection of *EGFR* mutations were 78.57%, 58.20%, and 63.41%, respectively. MD, mutation detected; MND, mutation not detected.

Supplementary Table. S8. Retrospective comparison study: correlation between ddEGFR and cobas EGFR test results.

Applied iQC index (*n = 171*)		cobas EGFR Test													
		**G719X**	**19del**	**T790M**	**E20Ins**	**S768I**	**L858R**	**L861Q**	**19del,T790M**	**G719X, S768I**	**G719X, L858R**	**G719X, L861Q**	**T790M, L858R**	**MND**	**Total**
**ddEGFR Test**	**G719X**	2													2
	**19del**		46												46
	**T790M**			0											0
	**E20Ins**				2									1	3
	**S768I**					0									0
	**L858R**						54							2	56
	**L861Q**							0						1	1
	**19del,T790M**		**3**						1						4
	**G719X,S768I**									3					3
	**G719X,L858R**						**1**				0				1
	**G719X,L861Q**	**1**										0			1
	**T790M,L858R**						**1**						3		4
	**MND**		2											48	50
	**Total**	3	51	0	2	0	56	0	1	3	0	0	3	52	**171**

κ coefficient = **0.9029** (95% C.I. 85.08–95.49%)

Detailed concordance rates of the retrospective comparison study group. Comparison between the ddEGFR and cobas EGFR test results, with application of only the iQC index criterion. MND, mutation not detected.

Supplementary Table. S9. Discordant analysis by Sanger sequencing in the retrospective comparison study group.

Sample No.	cobas EGFR test		ddEGFR test		ddEGFR MI		Sanger Seq
1	MND	-	MD	L858R	L858R, 25.47%		Invalid
2	MD	L858R	MD	L858R/T790M	L858R, 42.9%	T790M, 1.01%	L858R
3	MD	19del	MD	19del/T790M	19del, 12.79%	T790M, 0.96%	19del
4	MD	L858R	MD	L858R/G719X	L858R, 43.22%	G719X, 1.98%	Invalid
5	MD	19del	MD	19del/T790M	19del, 22.45%	T790M, 1.22%	19del
6	MD	19del	MD	19del/T790M	19del, 47.94%	T790M, 0.87%	19del
7	MD	19del	MND	-	-		WT
8	MND	-	MD	E20Ins	E20Ins, 14.55%		Invalid
9	MD	19del	MND	-	-		19del
10	MND	-	MD	L861Q	L861Q, 64.93%		L861Q
11	MD	G719X	MD	G719X/L861Q	G719X, 11.36%	L861Q, 13.64%	Invalid
12	MND	-	MD	L858R	L858R, 6.46%		Invalid

Based on the results of the retrospective comparison with the cobas EGFR test results, 12 samples were valid and discordant according to the ddEGFR test. Samples that gave discordant results between the cobas EGFR and ddEGFR tests were analyzed by Sanger sequencing. MD, mutation detected; MND, mutation not detected.

Supplementary Figures

Supplementary Figure. S1. Schematic of re-analysis of discordant samples

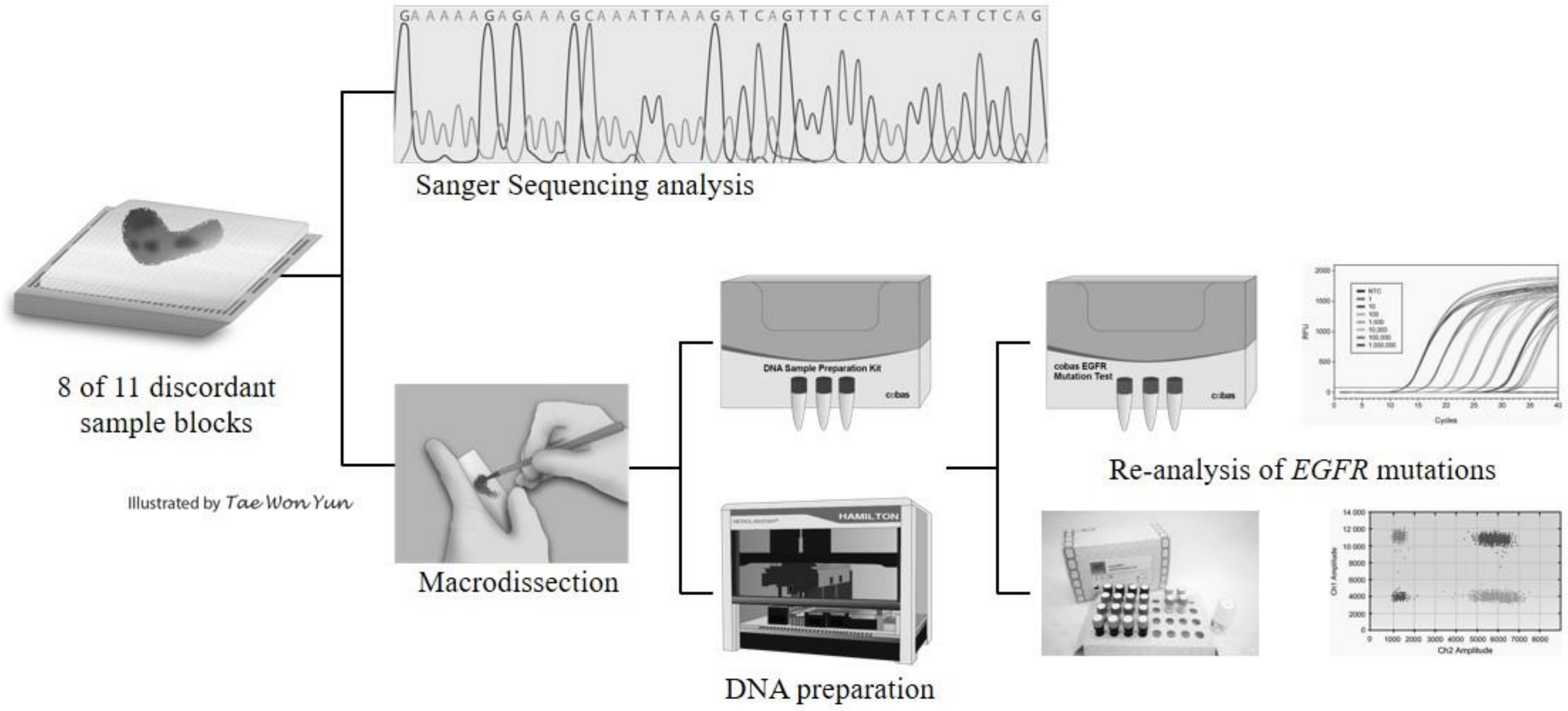

Eight of eleven discordant samples were verified by Sanger sequencing. In addition, after increasing the tumor-to-normal tissue ratio, *EGFR* mutations were re-analyzed using the cobas EGFR and ddEGFR tests.

Supplementary Figure. S2. Distributions of iQC index and DIN in the retrospective comparative study group

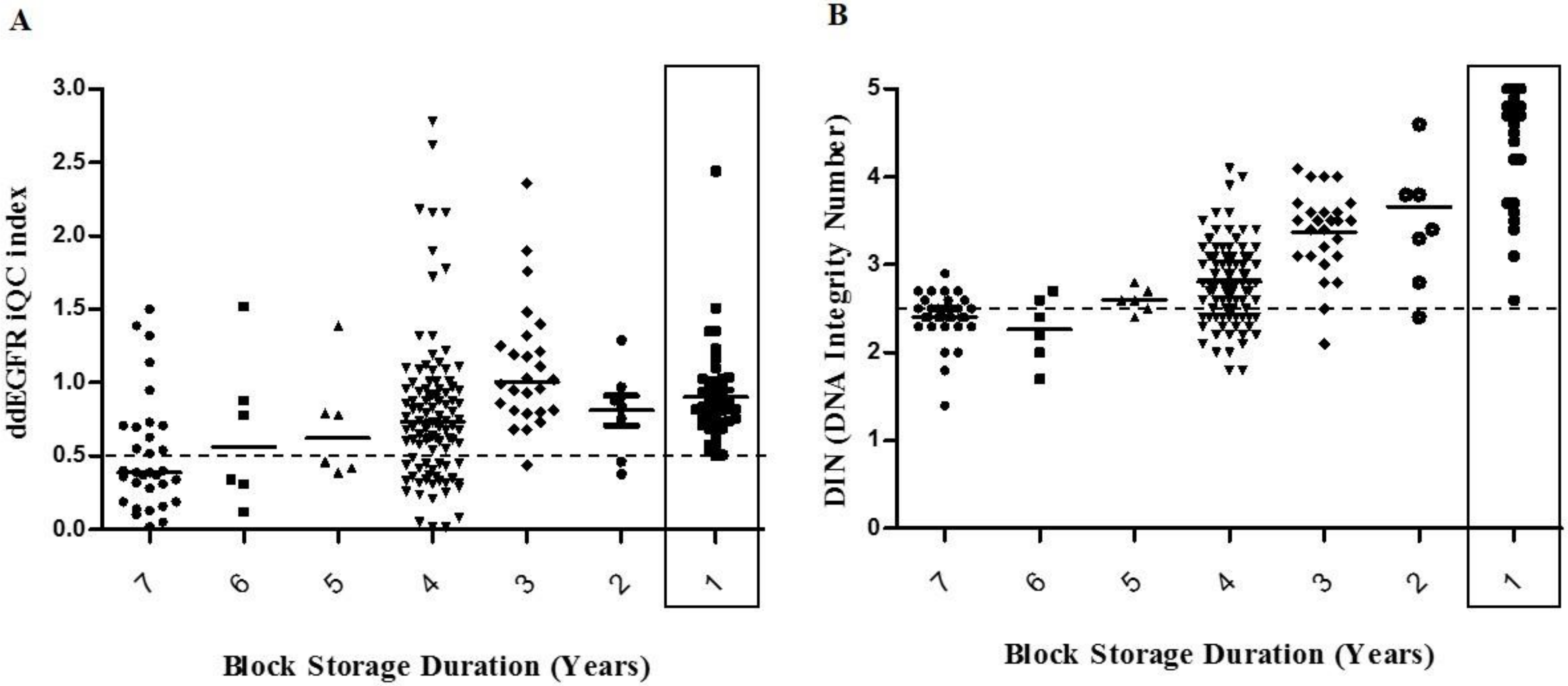

Distributions of iQC index (A) and DIN values (B) in the retrospective comparative study group. Most of the more recent samples (taken within 1 year) satisfied the sample criteria defined above (black dotted line).
